# Non-destructive crystallinity assessment of indomethacin in tablets made from smartFilms^®^ using terahertz time-domain spectroscopy

**DOI:** 10.1038/s41598-022-10041-1

**Published:** 2022-04-12

**Authors:** Jan Ornik, Lara Heidrich, Robert Schesny, Enrique Castro-Camus, Cornelia M. Keck, Martin Koch

**Affiliations:** 1 Department of Physics and Material Sciences Center, Philipps-Universität Marburg, Renthof 5, 35032 Marburg, Germany; 2 Department of Pharmaceutics and Biopharmaceutics, Philipps-Universität Marburg, Robert-Koch-Str. 4, 35037 Marburg, Germany; 3 Centro de Investigaciones en Optica A.C., Loma del Bosque 115, Lomas del Campestre, Leon, Guanajuato 37150 Mexico

**Keywords:** Drug delivery, Terahertz optics

## Abstract

We use terahertz (THz) time-domain spectroscopy (TDS) to assess the crystalline state of indomethacin (IM) when loaded in smartFilms®. We found that smartFilms favour the amorphous IM (A-IM) for low loading concentrations. For higher concentrations, IM recrystallizes in its $\alpha$- crystalline form and the amount of A-IM in the smartFilms reduces. Both, $\alpha$- and A-IM are preferred over the more common $\gamma$- crystalline form, as they exhibit better water solubility, which can increase the oral bioavailability of the drug.

## Introduction

The capacity of the body to assimilate many pharmaceutical substances is strongly dependent, not only on the molecular composition, but also on the state of aggregation of such molecules^1^. Substances orally administrated as tablets, are often in a crystalline form, which in general is a highly packed and relatively stable structure that does not always favour its water salvation in the digestive tract and subsequent assimilation by the body^2,3^. This is the case for indomethacin (IM), a non-steroidal anti-inflammatory class II drug according to the biopharmaceutics classification system (BCS)^4^. IM is commonly synthesized in a triclinic ($P\bar{1}$) lattice, also known as $\gamma$-IM. However, other forms such as the less stable monoclinic ($P2_1$) lattice known as $\alpha$-IM, and its amorphous form (A-IM) show better solubility in water and consequently better bioavailability during its transition through the digestive tract^2^.

In this article we demonstrate that dispersing IM in smartFilms® resulted in the formation of the A- as well as the $\alpha$-crystalline form of IM. SmartFilm-technology has been recently introduced and it aims at improving the bioavailability of poorly soluble pharmaceuticals by transforming and maintaining them in their amorphous state^5–7^. Our results also show that terahertz (THz) time-domain spectroscopy (TDS)^8^ can be used for quality control purposes through non-destructive inspection of the amorphousness and crystallinity of active pharmaceutical ingredients (API) in tablets.

## Results

### Indomethacin samples

**Figure 1:**
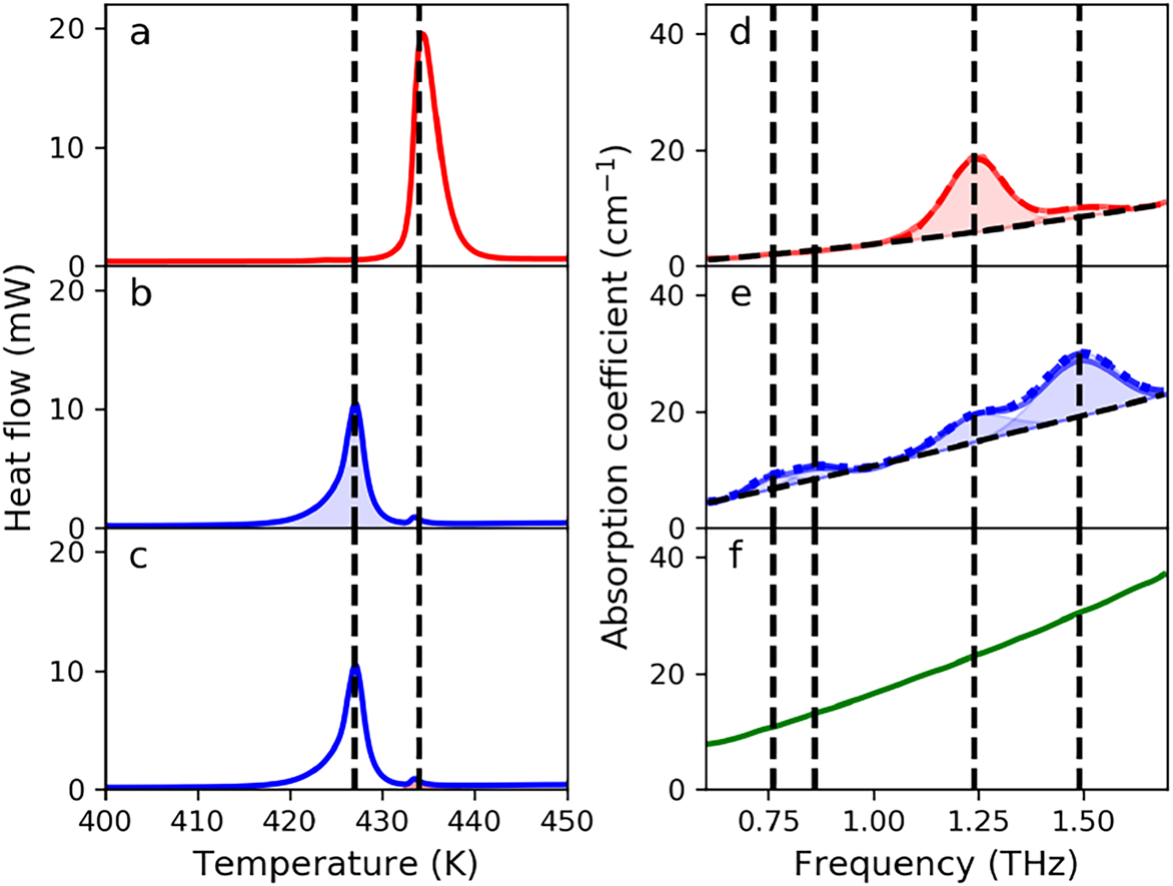
(**a**) DSC curve for $\gamma$-IM which shows a single peak at 434.3 K. (**b**,**c**) DSC curve for the recrystallized-IM which shows a strong peak at 427.0 K and a weak peak at 434.3 K. The area shaded in light blue in panel (**b**) corresponds to the $\alpha$-IM, while the area shaded in light red in panel (**c**) corresponds to the $\gamma$-IM present in the recrystallized sample. (**d**) THz absorption spectrum of $\gamma$-IM (red solid line) with fitted featureless background absorption (black dashed line), and two fitted characteristic absorption peaks (i.e., ${\hbox {G}}_{\gamma ,1}$ and ${\hbox {G}}_{\gamma ,2}$, shaded area). Dashed red line corresponds to the fitted spectrum. (**e**) THz absorption spectrum of the recrystallized-IM sample with fitted featureless background absorption (black dashed line), and four fitted characteristic absorption peaks (i.e., ${\hbox {G}}_{\alpha ,1}$-${\hbox {G}}_{\alpha ,4}$, shaded area). Additionally, the estimated $\alpha$-IM absorption is shown as a dotted blue line and the dashed blue line corresponds to the fitted spectrum. (**f**) THz absorption spectrum of the A-IM.

The $\gamma$-IM and the recrystallized-IM samples were characterized by DSC to determine whether there were more crystallographic forms in our samples. In the DSC curve of the $\gamma$-IM sample, shown in Fig. 1a, a single endothermic peak at 434.3 K was observed. This peak corresponds to the melting of the $\gamma$-IM^9^. The DSC curve for the recrystallized-sample, shown in Fig. 1b,c, showed a strong peak at 427.0 K, which corresponds to the $\alpha$-form^9^. Additionally, a smaller peak at approximately 434.3 K can be observed, which indicates that a small amount of $\gamma$-IM remained in the recrystallized-sample. From the area under each peak it was possible to determine that the recrystallized-IM contained approximately 94% of $\alpha$-IM and 6% of $\gamma$-IM.

The absorption spectra in the 0.6 THz to 1.7 THz range of $\gamma$-, recrystallized- and A-IM samples are shown in Fig. 1d–f, respectively. Since the DSC analysis revealed that the recrystallized sample consisted out of 94% $\alpha$-IM and 6% $\gamma$-IM, the absorption coefficient for the pure $\alpha$-IM was obtained by correcting the absorption coefficient of the recrystallized sample. This was done by first multiplying the values of the absorption coefficient of the $\gamma$-IM with 0.06 (i.e., proportion of $\gamma$-IM) and subtracting them from the values of the absorption coefficient of the recrystallized sample. The values obtained this way were subsequently divided by 0.94 (i.e., proportion of $\alpha$-IM) to obtain the estimation for the corrected THz absorption coefficient of the pure $\alpha$-form, which is shown as the blue dotted line in Fig. 1e.

In the investigated frequency range two distinct absorption peaks, a stronger peak at approximately 1.25 THz, and a weaker one at approximately 1.5 THz could be identified for the $\gamma$-form (Fig. 1d). This agrees with the previous reports^10,11^. These two absorption peaks could be observed in the $\alpha$-form (Fig. 1e) as well. However, in case of $\alpha$-form, the absorption peak at approximately 1.5 THz is stronger compared to the one at approximately 1.25 THz. Furthermore, two additional and comparably weaker peaks were observed at approximately 0.75 THz and 0.85 THz. The observed spectral features of the $\alpha$-form are in accordance with the reported absorption spectra^12,13^. On the contrary to the crystalline forms of IM, the absorption spectrum of the A-IM (Fig. 1f) shows a monotonically increasing trend without distinct spectral features, which has also been observed before^10,11^. Such featureless absorption spectrum is common in this frequency range for substances in amorphous state^14,15^.

The absorption spectra of the two crystalline forms were fitted by a combination of a second degree polynomial and a series of Gaussian functions, which were used to describe the number of the observed absorption peaks. The polynomial, however, was used to describe the featureless background absorption associated mainly to the scattering. The parameter describing the absorption peaks in terms of peak amplitude (*G*), frequency position of the peak ($\upsilon$_0_) and peak width ($\triangle \upsilon$), which were obtained by fitting the absorption spectra of the two polymorphs, are listed in Table 1.

**Table 1:** Gaussian peak parameter obtained from fitting the absorption spectra of $\gamma$- and $\alpha$-IM.

Peak	${G}$ (cm^–1^)	${\nu _0}$ (THz)	${\triangle \nu }$ (THz)
${\gamma _1}$	12.50	1.24	0.10
${\gamma _2}$	1.69	1.48	0.10
${\alpha _1}$	2.03	0.76	0.07
${\alpha _2}$	1.97	0.86	0.07
${\alpha _3}$	5.00	1.24	0.11
${\alpha _4}$	10.18	1.49	0.12

### SmartFilms and physical mixtures

**Figure 2:**
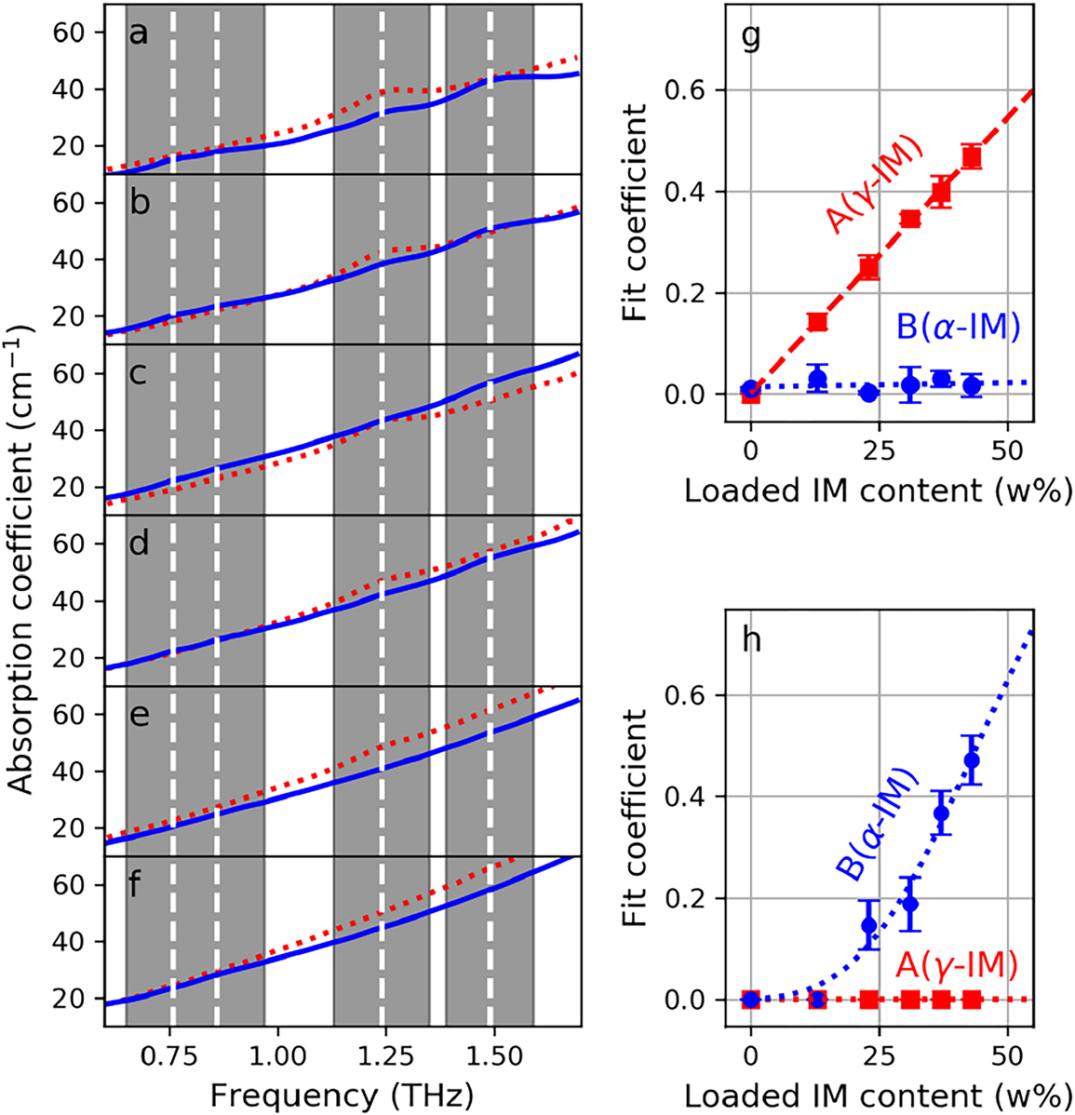
(**a**–**f**) show the spectra of the smartFilms (continuous blue) and physical mixtures (dotted red) samples for IM concentrations of 43, 37, 31, 23, 13, 0 w%, respectively. Fit coefficients A (red squares) and B (blue circles) as a function of concentration for physical mixture samples (**g**) and for smartFilm samples (**h**). Coefficients A and B correspond to the $\gamma$-IM and $\alpha$-IM, respectively. The dashed red line in (**g**) represents the linear fit and the dotted lines in (**g**–**h**) serve as a guide-to-the-eye.

The obtained parameter for the absorption peaks of $\gamma$- and $\alpha$-IM were used for the crystallinity assessment of IM in smartFilms and physical mixtures by describing the absorption spectra as a sum of background absorption and a linear combination of $\gamma$- and $\alpha$-IM absorption peaks. Thus, the following function was fitted to the absorption spectra:

1 $$\begin{aligned} f(\nu )=A \sum _{i=1}^{2} {G_{\gamma _i}e^{-\left( \frac{\nu -\nu _{0}^{\gamma _i}}{\triangle \nu _{\gamma _i}}\right) ^2}} + B \sum _{i=1}^{4} {G_{\alpha _i}e^{-\left( \frac{\nu -\nu _{0}^{\alpha _i}}{\triangle \nu _{\alpha _i}}\right) ^2}} + O(2), \end{aligned}$$

where $\nu$ is frequency, $G_{\gamma _i}$, $\nu _{0}^{\gamma _i}$, $\triangle \nu _{\gamma _i}$ are amplitude, frequency position and width of the *i*-th $\gamma$-peak, respectively, and $G_{\alpha _i}$, $\nu _{0}^{\alpha _i}$, $\triangle \nu _{\alpha _i}$ are amplitude, frequency position and width of the *i*-th $\alpha$-peak, respectively. The first and second summation term include $\gamma$ and $\alpha$ related Gaussian peaks, respectively, and *O*(2) is the second order polynomial, which is used to describe the featureless background absorption associated to the absorption and scattering of the paper matrix and the A-IM. The coefficient *A* and *B* are obtained from the fit and related to the content of $\gamma$- and $\alpha$-IM in the samples, respectively. The values of the two fit coefficient are shown in Fig. 2g,h for the physical mixture and smartFilm samples, respectively.

For physical mixtures (Fig. 2g), the coefficient *A* corresponding to the $\gamma$-IM content increases linearly with the content of IM, which is expected since the physical mixtures were prepared using the $\gamma$-form. On the contrary, the coefficient *B* corresponding to the amount of $\alpha$-IM remains constant and close to zero as expected, since this IM form was not added to the physical mixtures. The corresponding absorption spectra for physical mixtures (red dotted lines) shown in Fig. 2f-a reveal an increasing absorption peak at 1.24 THz, which is the most prominent absorption feature of the $\gamma$-IM. Furthermore, the most prominent absorption peak for $\alpha$-IM at 1.49 THz could not be observed, which was expected, since $\alpha$-form was not present in the physical mixtures.

For smartFilm samples (Fig. 2h), the coefficient *A* corresponding to the $\gamma$-IM on one hand, remains zero regardless the IM content in the samples. On the other hand, the coefficient *B* corresponding to the $\alpha$-IM, starts increasing non-linearly for IM content exceeding 13 w% and remains zero for lower content. In the corresponding absorption spectra (blue solid lines) in Fig. 2a–f all four peaks corresponding to the $\alpha$-form can be observed for samples with higher content (Fig. 2a–d). Furthermore, no absorption peaks could be observed either for the non-loaded (Fig. 2f) nor for the samples with the lowest loading (i.e., 13 w%, Fig. 2e), which indicates that the loaded IM remained amorphous in the smartFilms.

**Figure 3:**
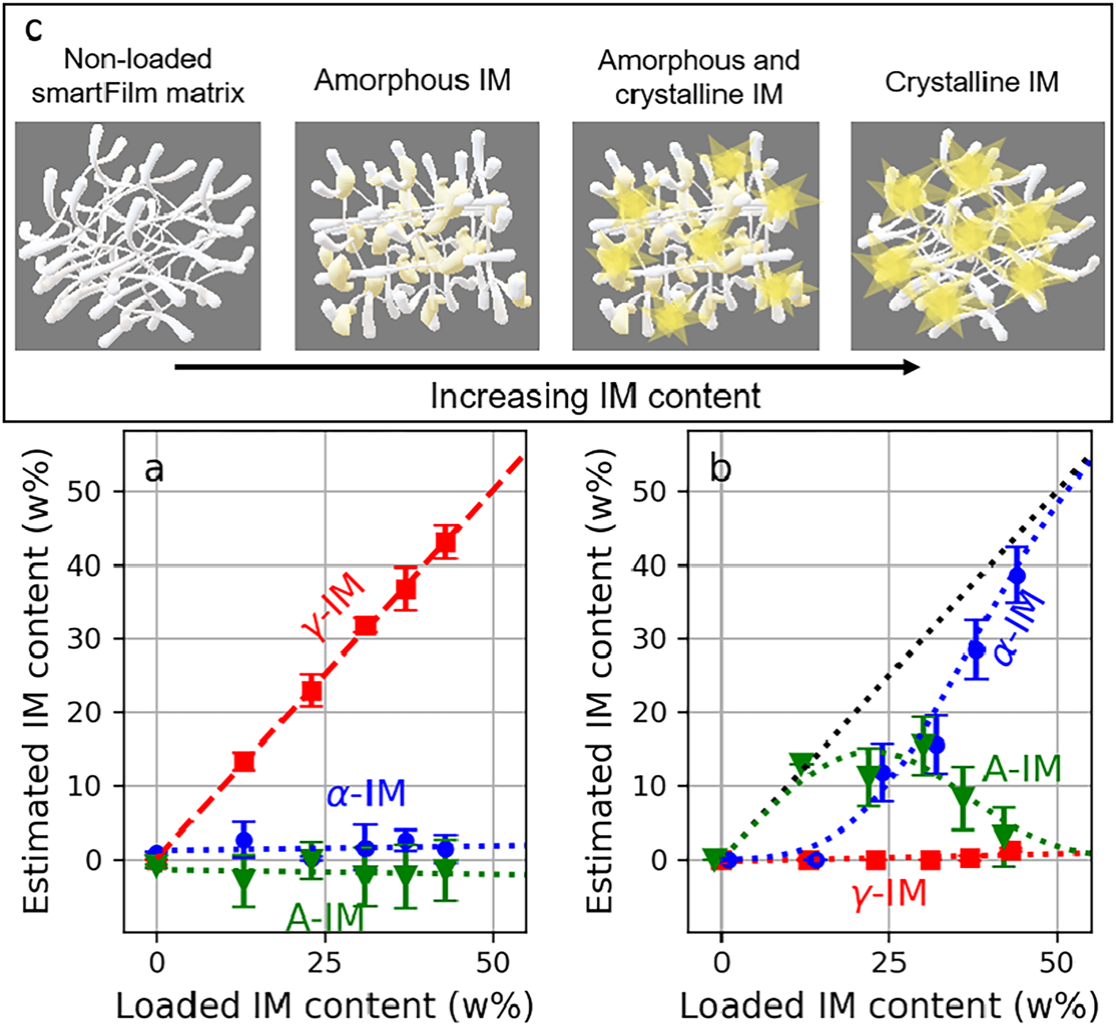
Estimated content of A- (green triangles), $\alpha$- (blue circles), and $\gamma$-IM (red squares) for the physical mixture samples (**a**) and the smartFilm samples (**b**). The red dashed line corresponds to the linear fit. All the dotted lines are provided as a guide-to-the-eye and represent no particular model. Additionally, for visualization purposes, the green and blue value markers in (**b**) were artificially shifted for +1% and -1% in the horizontal direction, respectively. (**c**) Schematic presentation of amorphous and crystalline IM loaded in the smartFilms.

To estimate the $\gamma$- and $\alpha$-IM content from the values of fit coefficient *A* and *B*, respectively, a conversion factor is needed. Since the physical mixtures contain only $\gamma$-IM, it is possible to obtain such an empirical conversion factor, which accounts for the contribution of the paper in the tablets, as well as other contributions such as scattering owing to variations in the porosity. We determine the conversion factor as the slope of linearly increasing *A* values (Fig. 2g), which equals to 0.0109 (w%)$^{-1}$. For each sample we divide the obtained *A* and *B* values by the obtained conversion factor to estimate $\gamma$- and $\alpha$-IM content, respectively. Once the content of the two crystalline forms is known, we assess the content of A-IM as the difference between the actual loading of the sample and the estimated $\gamma$- and $\alpha$-IM content. In other words, we assume that the remaining IM content (i.e., not $\gamma$- nor $\alpha$-IM) in the samples is amorphous. The estimations for the physical mixtures and smartFilms are shown in Fig. 3a,b, respectively.

In the case of the physical mixtures all IM remains in the $\gamma$-form, since the estimated content of A- and $\alpha$-IM remains zero within the error bars. This is expected as the physical mixtures contain only the $\gamma$-form. For the smartFilms the observed trend is very different. For low concentrations of IM the data suggests that all of the loaded IM was maintained in its amorphous state by the smartFilms. However, for higher loads, part of IM recrystallizes as $\alpha$-IM. Furthermore, the observed trend suggests that if IM is excessively loaded into the smartFilms, the amount of the A-IM in the tablets reduces and IM recrystallizes as $\alpha$-IM. Additionally, the formation of $\gamma$-IM is completely inhibited.

## Discussion

The results obtained are in line with previous studies and support the current understanding on how smartFilms can increase the bioavailability, i.e., the absorption of poorly water-soluble substances after oral application. An API is considered to have poor water solubility, if more than 1000 parts of solvent (i.e., water) are required for one part of solute (i.e., API). Today many new chemical entities are even less soluble and require more than 10,000 parts of solvent for one part of solute. Hence, 100 mg of such a compound would require more than 1000 g solvent. As this volume is not available in the gastro-intestinal tract, the compound will dissolve slowly and cannot be dissolved completely after oral application and thus cannot sufficiently be taken up by the body. In fact, the bioavailabilty of the compound will be insufficient. The problem can be overcome by increasing the dissolution rate and the kinetic solubility of the compound. Both properties can be increased by either transforming the compound from its crystalline state into an amorphous state or into a better soluble crystalline form, and/or by decreasing the particle size of the crystalline material.

The smartFilm-technology enables us to maintain a material that is normally in a crystalline form in its amorphous state^5–7^. The mechanism is not fully elucidated so far. From the data up to date it is suggested that the fibres and pores of the smartFilm-matrix, which are composed of cellulose, prevent the re-crystallization of the drug substance after evaporation of the solvent, either due to the small pore size which restricts the growth of crystals and/or due to interactions with the matrix fibres^6,7^. Within this picture, the number of pores of the smartFilm-matrix limits the maximum loading capacity^16,17^. Hence, a chemical compound can only be loaded into smartFilms in amorphous state until the pores are exhausted. Higher loads will result in re-crystallisation of the chemical compounds in the paper matrix (Fig. 3c).

In this study the maximum loading of A-IM in the smartFilm-matrix was approximately 15 w%. Higher loads resulted in crystallization of IM. Interestingly, the re-crystallization was not obtained in the thermodynamically more stable $\gamma$-form but in the less stable $\alpha$-form that possesses a higher solubility when compared to its more stable $\gamma$-form. Re-crystallization of the $\alpha$-form from different solvents or after different treatments has been reported in multiple previous studies and can have different causes^18–20^. Albeit the observed effect is considered to be unrelated to the smartFilm-matrix and caused, instead, by different crystallization phenomena that were not the focus of the present work and should be investigated in more detail in future studies.

## Conclusions

The fact that the $\alpha$- and $\gamma$- forms of IM show distinctive absorption peaks in the THz range allowed us to assess their presence in tablets prepared from smartFilms and from physical mixtures of $\gamma$-IM and paper matrix. Furthermore, it was possible to estimate the content of the two polymorphs. Our measurements suggest that the use of smartFilms promotes the formation of A-IM up to approximately 15 w% as well as formation of $\alpha$-IM, whereas $\gamma$-IM is not formed. This finding has two important implications. Firstly, it is possible to produce pharmaceutical tablets that contain a large proportion of A-IM as well as $\alpha$-IM. These two forms possess higher water solubility compared to the conventionally used $\gamma$-IM^1^, which can consequently improve the bioavailablity of the API. Secondly, THz TDS provides a sensitive non-destructive way to assess the amount of polymorphs present in the tablets. Therefore, it has the potential of being used as a quality control tool in the production of tablets made from smartFilms containing IM.

## Methods

### Sample preparation

Commercially available $\gamma$-IM (Acros Organics, 97.5%) was used without further purification. To obtain $\alpha$-IM we followed a method presented by Kaneniwa et al.^21^ by dissolving 1.5 g of $\gamma$-IM in 60 ml of ethanol. The solution was heated up to 75$^\circ$C and filtered warm using a funnel and a filter paper circle (LLG-Labware, diameter: 125 mm, pore size 5-13 $\mu$m). By subsequent addition of cold distilled water $\alpha$-IM precipitated and was removed by filtration and dried overnight in a vacuum desiccator over P$_2$O$_5$. Henceforth we refer to this sample as the recrystallized-IM. A-IM was prepared by melting $\gamma$-IM in a holder and subsequently quench-cooling the melt within the holder in liquid nitrogen. The sample retained a disk-like shape of the sample holder that it was contained in. Therefore, no pressing of the sample was needed. The $\gamma$-IM and the recrystallized-IM powders were pressed into tablets before the THz investigation. A hydraulic press (Model No. GF-10B Cl. 1.0, Enerpac) was used for compression with a force of 30 kN for 90 s.

For the smartFilm preparation ordinary coffee filter paper (“Original 1 × 6” naturbraun, Melitta) was used and cut into smaller circular pieces with a diameter of 60 mm. A stock solution (28.27 mg/ml) was prepared by dissolving $\gamma$-IM in an acetone (25 mol%)–ethanol (75 mol%) mixture, since the solubility of IM is increased when the two solvents are mixed in this specific ratio^18^. Afterwards 500 $\mu$l of the solution were applied into the middle of each filter paper. After drying, this led to a loading of 0.5 mg/cm$^2$ corresponding to 14.14 mg of IM per paper basis and loading cycle. For higher loads the procedure was repeated up to ten times. SmartFilms with five different loads were prepared in triplicate (1, 2, 3, 4, 5 mg/cm$^2$ corresponding to 13, 23, 31, 37, 43 w%, respectively). The smartFilms were cut into smaller pieces of approximately 16  mm by 16  mm in size and pressed into tablets with a force of 60 kN for 90 s.

As a positive control, physical mixtures were prepared by mixing pulverised filter paper and crystalline $\gamma$-IM in corresponding ratios to the smartFilm samples (13, 23, 31, 37, 43 w%). The physical mixtures were subsequently pressed into tablets with a force of 60 kN for 90 s. Additionally two types of paper tablets without addition of IM (0 w%) were prepared by pressing (60 kN for 90 s) either pulverized filter paper or pieces of filter paper.

### Differential scanning calorimetry (DSC)

DSC measurements were performed using a DSC 3 (Mettler Toledo, USA). 1–4 mg of sample powder in an aluminium pan were analysed in each measurement. The samples were heated up from 293 K to 473 K with a heating rate of 10 K/min.

### Terahertz time-domain spectroscopy

We used a fiber-coupled THz TDS system for this investigation. Further details about the system can be found in Ref.^22^. All measurements were performed in transmission under nitrogen atmosphere by recording the time-resolved waveform of a THz electromagnetic transient passing through the sample. For each sample measurement an additional reference measurement was performed by recording the waveform of a transient in the absence of a sample. The investigated samples had a form of bi-planar faceted tablets with a diameter of 13 mm. The thickness of tablets differed between 0.5 mm and 2 mm. The absorption spectra for all samples were calculated from the recorder THz waveforms by using the TeraLyzer software, which is based on algorithms presented in Refs.^23–25^.
